# Study protocol: a cluster randomized controlled trial to assess the effectiveness of a therapeutic educational program in oral health for persons with schizophrenia

**DOI:** 10.1186/s13033-016-0096-0

**Published:** 2016-10-05

**Authors:** Frederic Denis, Isabelle Millot, Nicolas Abello, Maud Carpentier, Audrey Peteuil, Agnès Soudry-Faure

**Affiliations:** 1 La Chartreuse Psychiatric Centre, 1, boulevard Chanoine Kir, BP 23314, 21033 Dijon Cedex, France; 2 Instance Régionale d’éducation et de promotion de la santé, 21000 Dijon Cedex, France; 3 Direction de la Recherche Clinique, University Hospital of Dijon, 21079 Dijon Cedex, France; 4 USMR-Réseau d’aide Méthodologiste, University Hospital of Dijon, 21079 Dijon Cedex, France

**Keywords:** Dental health, Oral health, Schizophrenia, Periodontal, Dental hygiene, Dental education

## Abstract

**Background:**

Schizophrenia is a severe mental disorder that affects 1 % of the world’s population, including 600,000 people in France. Persons with schizophrenia (PWS) have excess mortality (their life expectancy is reduced by 20 %) and excess morbidity. In addition, such persons may have a large number of missing or decayed teeth. Dental caries and periodontal measurement indexes are often twice as high as the level found in the general population. Poor oral health can also affect quality of life and oral health is inseparable from general health. The management of oral health problems needs a multidisciplinary approach. According to the World Health Organization, the aim of therapeutic education (TE) is to help patients take care of themselves and to improve empowerment and recovery. In this educational approach, it is important to take into account the patient’s personal experience. Though rarely investigated, the personal experience of PWS in oral health quality of life (OHRQoL) must be used to build a therapeutic educational programme in oral health (TEPOH) in a multidisciplinary approach, and the effectiveness of this program must be evaluated.

**Methods/design:**

We report the protocol of a randomized controlled cluster study. This study will be conducted in twelve hospitals in France. We hypothesized that a decrease of 20 % in the proportion of patients with CPI ≥ 3 would establish the effectiveness of TEPOH. Therefore, 12 hospitals will be randomly allocated to either TEPOH or no TEPOH. Altogether, they will have to recruit 230 PWS, who will be randomly allocated with a ratio of 1:1 to one of two conditions: control without intervention versus the group benefitting from TEPOH.

**Discussion:**

If successful, the study will generate methodologically sound results that provide knowledge on the effectiveness of a TEP in oral health for PWS. The results can be used to promote OHRQoL in a global health approach and develop appropriate strategies to encourage and facilitate financial support for healthcare, the multidisciplinary treatment of dental disorders, and the development of training in oral and mental health for caregivers.

*Trial registration* Clinical Trials Gov NCT02512367. Date registered 19 July, 2015

## Background

Schizophrenia is a severe mental disorder that affects 1 % of the world’s population [1, 2], including 600,000 people in France. Schizophrenia is characterized by a set of different symptoms varying in intensity: the most dramatic are delusions, hallucinations, mental dissociation, and, for the most handicapping, social withdrawal, denial of the body, and cognitive difficulties [3]. Persons with schizophrenia (PWS) have excess mortality (their life expectancy is reduced by 20 %) and excess morbidity [4, 5]. Among somatic comorbidities in PWS, poor oral health has been reported by many authors and contributes to the overall poor health of these patients [6–8]. Generally, the symptoms of schizophrenia lead to disturbances in the progression of thought, errors in contextual analysis and errors of logic. Often, PWS do not recognize their health needs and delay seeking advice or treatment [9].

This is the case for all related somatic disorders that, by lack of analysis inherent to this disease, prevent the persons from recognising the condition or cause them not to make the right decisions to solve problems independently [9, 10]. Moreover, difficult relationships with professional caregivers (fear of mental illness, lack of training) and the health system in general (difficulties in gaining access to private practice, environment, cost…) are additional obstacles contributing to deficient somatic care [11, 12].

One of the most visible elements of poor oral health is edentulousness, and a large number of missing or decayed teeth (leading to pain, infection, masticatory and digestive problems) can be noticed in this population [13, 14]. Dental caries, periodontal or infectious diseases on the one hand, and metabolic disturbances induced by antipsychotic treatments (diabetes, obesity, xerostomia…), poor diet and lifestyle behaviours (diet rich in sugars, use of psychoactive substances such as tobacco, and inadequate oral hygiene), all combine to lead to poor health [14–16].

Generally, negative symptoms, age, duration of mental illness, xerostomia, low socio-economic and cultural determinants are risk factors common to tooth decay and periodontal disease [17–21]. These factors are aggravated by stigmatization and discrimination, which is why PWS do not have the same amount of attention, in terms of their physical health, as others [22, 23]. International data confirm that oral health is poor in PWS. Dental caries and periodontal measurement indexes are often twice the level found in the general population [8, 14, 20, 21]. Generally speaking, less than 10 % of the population of industrialized countries suffer from severe forms of periodontitis [24, 25]. Periodontal health is assessed using different indexes. The periodontal index, called the CPI (community periodontal index), is the World Health Organization’s (WHO) reference index [26]. It is estimated that 40 % of schizophrenics exhibit a CPI ≥ 3 according to the literature [27, 28]. A CPI ≥ 3 indicates advanced periodontal disease, with a loss of alveolar bone depending on severity. The most consistent predictors of periodontal disease are age, education, income, smoking status, dental visits, the number of remaining teeth, the number of decayed coronal surfaces, the number of decayed root surfaces and diabetes. Periodontal disease is therefore influenced by medical, social and behavioral factors [29, 30]. Appropriate treatment to control periodontal infection can limit the number of affected sites and stop the progression of the disease [31]. For the management of oral health problems, it is important to control the frequency of daily brushing and the side effects of antipsychotic treatments, to ensure regular dental visits and to monitor the respect of dental hygiene advice [32].

Poor oral health can also affect quality of life through the social and psychological impact of the deterioration in smile aesthetics for self-esteem, and self-confidence [8, 22]. The oral side effects of antipsychotics generally include a reduction in the salivary flow rate. Conversely, Clozapine can induce hypersalivation. However, a dry mouth was the chief complaint among 40 % of the psychiatric patients while dental pain was the main complaint among 60 % of the control group [33].

There are also the neurological effects of first-generation antipsychotics (FGAs) (dystonia, dyskinesia), which produce shaking and prevent effective brushing, alter chewing and swallowing [34, 35]. Second-generation antipsychotics (SGAs) induce more metabolic side effects and fewer neurological effects [36, 37]. Evidence of a relationship between metabolic disorders and oral deficiencies has gradually grown over the last 10 years with, on the one hand, increased knowledge on the pathophysiology of the syndrome and its consequences on cells and tissues and, on the other hand, the observation that certain infectious diseases of the mouth, such as periodontal disease, have already been associated with each of the components of the metabolic syndrome [38]. This means that diabetes is a risk factor for the development of periodontal disease. Control of periodontal infection would furthermore contribute to controlling diabetes [39–41]. Periodontal disease is also a risk factor for cardiovascular diseases (ischemic heart disease) and associated with excess mortality in PWS. One explanation is that poor oral hygiene allows oral bacteria to enter the bloodstream. Immune complexes are then formed, which, in turn, elicit inflammatory responses in arteries [42, 43]. Furthermore, oral health is not just about having healthy teeth, it is a ‘standard of health of the oral and related tissues which enables an individual to eat, speak and socialise without active disease, discomfort or embarrassment and which contributes to general well-being’. Oral health is thus inseparable from general health, and managing oral health problems needs a multidisciplinary approach. Currently, oral health prevention and promotion programs, based on knowledge in the general population and transposed for persons with severe mental disorders, have not proven to be very effective [44–46]. The particular profile of persons suffering from schizophrenia must be taken into account. Khokhar et al. [45] pointed out that there were no studies with a high enough level of proof to support current practices in this field.

The research deals with ways to help persons with smoking, a sedentary lifestyle, unbalanced diet, and dental health status [47–49]. This requires people, especially the family circle, social, medical and social services personnel, private nurses and general practitioners [50–52], to be trained to interact frequently with such patients. This training is particularly necessary because healthcare professionals often have an aversion to these patients (fear of the mental illness, lack of training), leading to numerous biases and generating an unhelpful attitude. The lack of initial training in this field contributes to misunderstanding among caregivers and the stigmatization of patients suffering from schizophrenia [12, 52]. It is therefore important not only to build awareness of oral health problems in PWS, but also to train these caregivers in psychiatric diseases. The aim of this training would be to foster better understanding of patients’ symptoms in order to avoid misunderstandings regarding their behaviour.

In response to the increased prevalence of chronic diseases, the very high rate of treatment non-observance, and the need for more personal autonomy, therapeutic education (TE) with small groups (five or six participants) is gradually emerging as a healthcare approach [53, 54].

For example, Lindenmayer et al., showed the effectiveness a structured wellness program using a psychoeducational curriculum for weight reduction and other metabolic markers in a large inpatient sample (275 patients with severe mental illness) [55].

According to the World Health Organization (WHO), the aim of TE is to help patients take care of themselves and to improve empowerment and recovery [54]. In this educational approach, it is important to take into account the experiences of persons taking part in multidisciplinary group learning. In a preliminary study, we confirmed the acceptability and feasibility of partnerships with PWS to develop an interactive guide to improve access to primary care providers for chronic disease management and health promotion [56]. TE is too often limited to cognitive information or movement training. Health beliefs are often not studied and the presentation of the causal links between inadequate behaviour and chronic disease is largely insufficient to treat a patient successfully over the long term [57, 58]. A new approach to TE programs depends on a specific environment to foster motivational behaviour change, not only “focused” on patients but developed with them. In this model, the person is the best expert of the disease. In the TE concept, as participants themselves are trying to learn, one can explore not only what the participants are talking about, but also how they are trying to understand and conceptualize the issue under discussion [59, 60].

Rarely investigated, the PWS experience in oral health quality of life (OHRQoL), must be used to build an educational therapeutic program in oral health in a small multidisciplinary group learning process.

## Aims

The principal objective of this study is to evaluate the effectiveness of a therapeutic education programme in oral health (TEPOH) for PWS. This aim will be achieved in the context of a randomized controlled cluster study using a population with schizophrenia sample recruited from out- or in-patients in psychiatric hospitals in France.

We hypothesize that a multidisciplinary therapeutic education program (TEP), involving dentists, psychiatrists, nurses, doctors, psychologists, PWS, caregivers and specialists in therapeutic education, will promote clinical improvements in oral health. In addition, we will assess TEPOH as a new care support strategy for improving the oral health related quality of life (OHRQoL) of PWS. We will also assess caregiver’s behavioural changes concerning oral health in PWS.

## Methods/design

It is a multicentre, cluster randomized controlled trial designed to assess the effectiveness of a TEPOH on PWS. Therefore, 12 hospitals will be randomly allocated to four clusters (a cluster is composed of three hospitals) where benefiting from TEPOH is available or not, and 230 PWS will be recruited and randomly allocated with a ratio of 1:1 to one of two conditions: control without intervention versus the group benefitting from TEPOH.

### Study process (Fig. 1)

#### Preliminary stage

Building a therapeutic educational program in oral health (Fig. 2). A preliminary phase of this study will consist of the creation of the program content. An expert group made up of health professionals, stabilized PWS, and carers of PWS has been created. A focus group (FG) led by a specialist in TE “Instance Régionale d’éducation et de promotion de la santé” (IREPS) explored the needs and expectations in oral health among PWS [61]. Participants were able to defend their priorities, preferences, values or experiences. The FG method is generally used to collect opinions, beliefs and attitudes about a topic or issue and to encourage the discussion of particular problems [62, 63]. After three FG targeted on oral health, problems in PWS were identified and summarized in the form of a questionnaire. These semi-structured questionnaires were presented by the same methodology to two distinct groups (composed of different people of the expert group): A group composed of health professionals only and a group of PWS and carers of patients. After a processing step of collecting information, the expert groups met again to validate the themes chosen for the construction of TEPOH program and the educational tools necessary for its implementation.

**Fig. 1:**
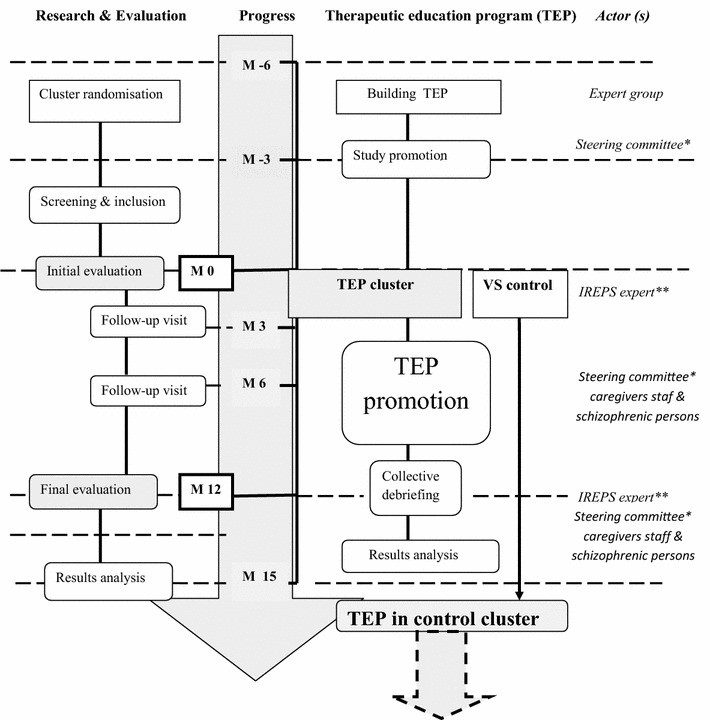
Study flow diagram. *Asterisk* 12 co-investigators, members of the expert group, a methodologist and statistician. *Double asterisk* Instances régionales d ‘éducation et de la promotion de la santé

**Fig. 2:**
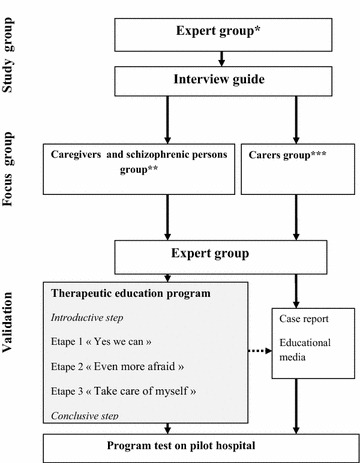
Therapeutic education program flow. *Asterisk* two dentists, three schizophrenic persons stabilized from a psychiatric viewpoint, one psychologist, one psychiatrist, two mental health nurses, one doctor, 2 patient aides. *Double asterisk* three schizophrenic persons, three accompanying patients. *Triple asterisk* one doctor, one dentist, one psychlogist, two nurses, one psychiatrist

The different chosen themes are:

1. Mobilization of motivational approaches by improving self-esteem and well-being, entitled: « Yes we can »
2. Demystifying the dental surgery entitled: « Even more afraid »
3. Improvement of oral health by a transverse approach (stop smoking, control diabetes, management of good diet…) to quality of life entitled: « Take care of myself »

Ultimately, the TE program will consist of three workshops, an introductory session and a debriefing session each lasting 90 min, spaced 2 weeks apart: A report form to allow patients to collect their behaviour changes with respect to oral health (improving oral hygiene, reducing tobacco use, attitudes of caregivers and others…) will be given to participants.

Before the three phases of the TEPOH, an introductory session will be conducted. It will consist of a presentation of the TEPOH and an exploration of OHRQol representations. It will be performed collectively with participating patients in the presence of their accompanying carers. Information will be collected using the metaplan method [64]. At the end of the TEPOH sessions, a second meeting will be conducted in order to carry out a comparative qualitative analysis with participating patients in the presence of their accompanying carers.

Finally, the time required for the TEPOH will be 7.5 h not counting the time for the baseline and follow-up assessments. Finally, the program will last 2 months.

This program has been designed for groups of five or six participants. The entire program will be pretested before its initiation according to the clinical study protocol on a pilot institution with a cluster not involved in the study, to assessing its feasibility and to make adjustments if necessary.

### Sample

#### Eligibility

Table 1 summarizes the criteria used to determine the eligibility of participants. A potential participant must have been diagnosed with schizophrenia as defined in the Diagnostic and Statistical Manual of Mental Disorders-Fifth edition (DSM-5) [65] and be receiving in- or outpatient care in one of the hospitals taking part in the study. Potential participants, over 18 years of age, can be of either sex. Schizophrenia is a severe mental disorder characterized by a set of different symptoms varying in intensity [3]. Persons not stabilized from a psychiatric viewpoint will be excluded. Persons will be excluded if hospitalized under stress or the person does not perceive the significance of the study or is not motivated to take care of their oral health. We will exclude people with severe somatic disorders regarding the aims of the study so as not to generate management problems of health care. Finally, we will exclude edentulous persons, because the absence of teeth is a limitation of our TEPOH for our end-point (periodontal disease) and temporarily for persons who have not updated their social rights to cover the cost of medical and dental care. In France, medical and dental care costs are covered by national health insurance and complementary health insurance or by universal health insurance (CMU) depending on the level of income. For people with low incomes, below 7771€ per year, CMU is free [66]. Indeed, as the aim of the program is to motivate participants to improve their oral health, if a participant does not have financial resources to access the health care system, the programme will be counterproductive. PWS have less access to dental care because of its cost. This problem must be solved before their inclusion in the study.

**Table 1:** Inclusion and exclusion criteria

Inclusion criteria	Exclusion criteria
Persons who have provided consent	Persons not covered by national health insurance
Persons of either sex over 18 years of age	Persons not stabilized from a psychiatric viewpoint or persons in an acute psychiatric episode
Persons with diagnosis of schizophrenia as defined in the Diagnostic and Statistical Manual of Mental Disorders-Fifth edition (DSM-5)	Pregnant or breast-feeding women Edentulous persons Persons hospitalized under stress
Receiving care in hospital (in- or outpatient)	Cannot understand or have a poor understanding of French Patients with risk of infective endocarditis^a^ or major risk of superinfection People undergoing chemotherapy

^a^Persons with prosthetic valve, cyanotic congenital heart disease, history of infectious endocarditis

#### Recruitment procedures

In 1960, France opted for mental health care based on the definition of local catchment areas. The country was divided into sectors of approximately 70,000 inhabitants [67]. Each sector is run by a reference hospital and a multidisciplinary team intended to provide preventive, as well as curative and rehabilitative care for all those within the catchment area who need it. Care is provided as close to home as possible. Patients can only have ongoing contact with the psychiatric sector’s outpatient unit and live in the community or can be hospitalized depending on severity their mental disorders [67]. The 12 hospitals that will take part in the study cover 42 psychiatric sectors. There are potentially 2,940,000 inhabitants and about 29,400 PWS for recruitment in the study. The local catchment areas of the 12 psychiatric hospitals are sufficiently distant from each other. The two closest hospitals are 60 kms apart, and the farthest are 600 kms apart. The 12 hospitals participating in the study are spread over the eastern half of France.

To maximize recruitment, co-investigators will provide information on the study in the different hospitals. They will inform potential participants about the study by means of the internal communication system for caregivers of the institution (e-mail or newsletter) and via the distribution of flyers informing PWS about the study. Those who express an interest will be given details of the study and screened for their eligibility by the study team of the co-investigator. All participants (caregivers and PWS) will be aware that the study relates to an assessment of the effectiveness of TEPOH and the exact nature of the research project (i.e., the existence of the intervention and control groups and the comparison of outcomes between them). Upon debriefing at the end of the study, the PWS who were in the no-TEPOH group could benefit from TEPOH.

Participants may withdraw from the trial either at their own request or at the discretion of the Investigator (acute psychiatric disorders). Participants will be made aware that withdrawal will not affect their future care. All participants (PWS and caregivers) will be made aware (via the information sheet and consent forms) that should they withdraw, the data collected to date cannot be erased and may still be used in the final analysis.

#### Procedure

*In the first phase*, hospitals will be randomized and groups of five or six participants will be organized. Randomization will be collective between the hospitals and not between patients. The local catchment areas of the 12 psychiatric hospitals are sufficiently distant from each other to avoid a bias of contamination between individuals of the same hospital. The trial will be conducted in the open because it is not possible to assess a person’s oral health without the person and the operator knowing. Cluster randomization is recommended by the “la Haute Autorité de Santé” (HAS) in France for evaluation trials of interventions to improve practices.

*In the second phase*, patients able to participate in the study will be screened in each local catchment area. Consenting participants will be contacted to arrange an oral health assessment. Evaluations will be conducted at the outpatient unit or at the hospitals where the patients are followed. Prior to arriving in their TEPOH group, they will be asked to complete a battery of psychological and OHRQoL questionnaires. Participants’ socio-economic status (SES) will also be collected.

*In the third phase*, participants will undergo a battery of tests to assess their oral health disorders using clinical and radiological examinations. At the end of this step, the TEPOH will be performed or not depending on the cluster to which the PWS belongs.

Before the start of the TEPOH, the teams of caregivers for the patients will be invited to participate in a focus group interview to assess their knowledge of oral health and their practices in the management in OHrQOL with PWS.

*The fourth phase* will consist of an evaluation at 3 months, 6 months and at 1 year after the initiation of TEPOH. All participants (patients and caregivers) will be asked to complete the same battery of tests they completed at baseline except for radiological examinations and SES.

The interviews for the OHRQoL will be managed to ensure that participants in the two groups (control vs TEPOH group) do not interact with each other.

#### Interventions

All participants at the twelve sites will be interviewed and clinically examined by the co-investigators.

Demographic and medical variables were extracted from institutional medical records: diabetic or not, prescribed drugs at the time of the examination, duration of mental illness. Other variables, namely the level of education, residential area, smoking habits, tooth brushing frequency and last dental visit, were collected directly by questioning the patient. Each participant will be asked to complete the French version of global oral health assessment index (GOHAI) [68], the schizophrenia quality of life questionnaire (S-QOL) [69], the Beck Depression Inventory-(BDI-II) [70], the stability status of symptom severity scale (PANSS) [71] and to answer other questions relating to their SES, health status (duration of mental illness), dental attendance and oral behaviour (smoking habits, tooth brushing frequency).

In a second time, each person will undergo a clinical oral examination in the dental surgery of the hospitals participating in the study. Pre-packaged and disposable instruments—dental mirrors, tweezers, 0.5 mm ball-ended CPITN probes (3.5–5.5 mm)—will be used and cotton rolls will be used to remove plaque. Caries will be assessed at the dentinal (D3) level using the DMFT index [72]. Dental plaque and calculus will be evaluated using the simplified oral hygiene index (OHI-S) [73] and periodontal disease by the community periodontal index of treatment needs (CPITN) [26].

This investigation will require 1 h for each participant. At the end of this evaluation, the TEPOH could start, as described previously, in all clusters selected for the study.

The mental health of PWS will be closely monitored by their care teams, and care co-investigators will be asked to inform the trial team of any adverse event that either the care co-ordinator or patient notices. The trial team will determine the seriousness and causality in conjunction with treating medical practitioners. Serious adverse events are divided into two categories, those that seem likely to be independent of TEPOH and those connected with TEPOH. All adverse events will be recorded and closely monitored until resolution, stabilization, or until it has been shown by the data monitoring committee that involvement in the trial is not the cause. All treatment-related serious adverse events will be recorded and reported to the Research Ethics Committee (REC) as part of the annual reports. Unexpected serious adverse events will be reported to the REC by the chief investigator, who will take appropriate medical action, which may include halting the trial and informing the sponsor of such action. Any participant who experiences an adverse event may be withdrawn from the study at the discretion of the investigator.

#### Control

Participants assigned to the control condition will be advised to maintain their existing lifestyles or take on new activities as they see fit. They will be examined only at the clinical evaluation at the start of the study and at 3, 6 and 12 months. For ethical reasons, if the program shows its effectiveness, it will be made available to all groups at the end of the study.

#### Outcomes

*Our primary end*-*point* will be the impact on periodontal disease. We have chosen periodontal index for our primary outcomes. Indeed, periodontal diseases are in relation with predictor in oral health (medical and dental condition, behavioural and SES) [29, 30].

The community periodontal index of treatment needs (CPITN) will be used to assess the periodontal status of the participants in the survey [26]. With the help of a World Health Organization periodontal probe, the depth of the periodontal pocket will be measured at six points on both the buccal (facial) and (lingual or palatal) surfaces of the index teeth. This measure will be used to score periodontal status and assess patients’ treatment needs. The initial sign of periodontal disease is bleeding of the gums. As it progresses, the gum retracts from the root surface of the tooth to form pockets. The deeper the pocket, the more severe the periodontal disease. The CPITN is a 5-point scale graduated as follows: no sign of periodontal disease (0), gingival bleeding after gentle probing (1), supragingival or subgingival calculus (2), pathological pockets 4–5 mm deep (3) and pathological pockets >5 mm deep (4).

*The secondary end point* will be impact on caries measured by the DMFT score [72]. Decay will be assessed using this standardized index to evaluate existing or past dental caries with scores ranging from 0 to 32. This examination will assess dental caries, dental restorations, fissure sealants, fixed prostheses, missing teeth and an additional category for traumatized incisors. High scores indicate worse dental health [72]. Root caries will not be assessed in this study.

*Other secondary evaluation criteria* will include a hygiene index. The debris index component of the simplified oral hygiene index (OHI-S) [73]. This tool will be used to measure the amount of debris. It will be scored on six tooth surfaces per participant.

For all of the dental examinations above, the dental specialists have been calibrated against the chief investigator, through repeated examinations of a separate pilot sample using similar indices, followed by meetings to discuss discrepancies and standardize procedures. Kappa scores of 0.9 for inter-rater agreement were achieved.

The following four tools will be used in this study: The OHRQoL from the French version of the self-assessment GOHAI scale [68]; schizophrenia quality of life from S-QoL [69]; The stability status of symptom severity from the PANSS scale [71] and depressive symptoms of schizophrenic persons from the Beck Depression Inventory scale [70].

Finally, a panoramic radiograph will be done if the patient does not have radiographs dating back less than 3 months. The dental panoramic film (orthopantomogram) is to detect periodontal disease, fractures, tumours and bone disorders from teeth and conditions that may affect the jaw and sinuses. A qualitative analysis of data collected during the TEPOH will make it possible to determine the evolution of representations, knowledge, attitudes and the practices of patients and professionals, and the ability of patients and professionals to assimilate an educational process.

### Sample/randomization and data-management

The literature highlights that up to 40 % of PWS disorders have a mean CPI ≥ 3 [27, 28], compared with less than 10 % in general population [24, 25]. Anticipating that the TEPOH will lead to a mean 20 % reduction in the CPI index of patients, taking into account cluster variability (CV = 0.37) and the intra-class correlation coefficient (CCI = 0.01), we will have to recruit a total of 202 patients to highlight a statistically significant difference between the two groups with a type-I error of 5 % and type-II error of 20 %.

Randomization will be done using SAS version 9.3 (SAS Institute Inc) by the team of statisticians of the Methodological Support Unit, Direction of Clinical Research, UHD, France. All participating hospitals will be randomly allocated to TEPOH or not using a random stratified sampling method depending on their location (urban/mixed/extra-urban) and their level of activity (number of patients per year). All data of included persons will be collected in an electronic case report form (CRF), created with the software CleanWeb™. Real-time likelihood and coherency tests will be implemented to check data entry with predefined rules in collaboration with all the co-investigators. Periodically, coherence reports will be sent to investigators with the aim to correct any highlighted errors. The database will be frozen only when all of the errors have been corrected and when no more are found.

### Statistical analysis

The interview data will be transcribed and coded using NVivo software to facilitate analysis of the factors identified by participants as affecting their attitudes and behaviours relating to changes in perception in OHRQoL. This process will yield a comprehensive account of the relevant barriers, motivators, and facilitators of OHRQoL. Among the intervention group participants, changes in attitudes will be documented. Results for qualitative covariates will be expressed as proportions. Quantitative variables will be expressed as means ± standard deviations (SD) when normally distributed, or as medians and ranges in other cases. Comparison of persons’ characteristics between a group of interest and the general population of PWS will be performed using Student’s *t* test, analysis of variance, Kruskal–Wallis non-parametric tests and Pearson’s Chi square or Fisher’s exact test when appropriate. A value of p < 0.05 will be considered statistically significant. All analyses will be performed using SAS version 9.3 (SAS Institute INC.).

Electronic data will be stored on secure data servers and hard copy materials will be retained in locked filing cabinets of Dijon University Hospital.

## Discussion

### Innovation

This document provides a complete description of the construction, the use and evaluation of the effectiveness of TEPOH specifically designed for PWS with their active participation for the first time.

The current study is innovative because it proposes to investigate the impact of a strong partnership between PWS and their caregivers to improve their oral health in France.

This TEPOH can be used to promote OHRQoL in a global health approach and to develop appropriate strategies to encourage and facilitate financial support for healthcare, the multidisciplinary treatment of dental disorders, prospective support for patients, and the development of training in oral or mental health for caregivers.

## Limitations

The active participation of patients is a key component of the study. However, the major difficulties with the inclusion of PWS in a long-term protocol study is that many PWS may be unable to cooperate due to their psychiatric illness, or lost to follow-up, or die during the study.

Secondly, although oral health has an impact on general health, self-esteem and quality of life often have a low priority in persons with psychiatric diseases in France. Furthermore, the somatic care of patients undergoing psychiatric treatment remains heterogeneous.

In this case, a key component will be the participation and involvement of healthcare teams in introducing TEPOH in their practices and in promoting TEPOH in all units of the hospital.

### Impact

Finally, this study is needed to inform the development of public policy and interventions that have the potential to improve the OHRQoL of PWS and the feasibility to engage PWS as full partners in TEPOH.

## Trial status

The preliminary phase of the study is completed and data collection for patients is closed. We will proceed with randomization of the clusters and will include the first patients in the study during July, 2016.

## Abbreviations

PWS: persons with schizophrenia
WHO: World Health Organization
TE: therapeutic educational
TEC: therapeutic educational concept
OHRQoL: oral health quality of life
FGAs: first-generation antipsychotics
SGAs: second-generation antipsychotics
TEPOH: therapeutic education program in oral health
CPP: committee for the protection of persons
FG: focus group technique
IREPS: Instance Régionale d’Education et de Promotion de la Santé”
DSM-5: Diagnostic and Statistical Manual of Mental Disorders-Fifth edition
CRF: case report form
HAS: Haute Autorité de Santé
GOHAI: global oral health assessment index
CV: cluster variability
ICC: intra class correlation
S-QOL: schizophrenia quality of life questionnaire
BDI-II: Beck Depression Inventory-II
DMFT: decayed missing filled teeth
SES: socio-economic status
OHI-S: simplified oral hygiene index
CPITN: community periodontal index of treatment needs
REC: Research Ethics Committee
PANSS: positive and negative syndrome scale
SD: standard deviation
